# Allele Summation of Diabetes Risk Genes Predicts Impaired Glucose Tolerance in Female and Obese Individuals

**DOI:** 10.1371/journal.pone.0038224

**Published:** 2012-06-29

**Authors:** Katarzyna Linder, Robert Wagner, Erifili Hatziagelaki, Caroline Ketterer, Martin Heni, Fausto Machicao, Norbert Stefan, Harald Staiger, Hans-Ulrich Häring, Andreas Fritsche

**Affiliations:** 1 Department of Internal Medicine, Division of Endocrinology, Diabetology, Nephrology, Vascular Disease and Clinical Chemistry, Eberhard Karls University Tübingen, Member of the German Centre for Diabetes Research, Tübingen, Germany; 2 2nd Department of Internal Medicine, Research Institute and Diabetes Center, Athens University Medical School, “Attikon” University Hospital, Athens, Greece; 3 Department of Internal Medicine, Division of Nutritional and Preventive Medicine, University Hospital Tübingen, Tübingen, Germany; John Hopkins Bloomberg School of Public Health, United States of America

## Abstract

**Introduction:**

Single nucleotide polymorphisms (SNPs) in approximately 40 genes have been associated with an increased risk for type 2 diabetes (T2D) in genome-wide association studies. It is not known whether a similar genetic impact on the risk of prediabetes (impaired glucose tolerance [IGT] or impaired fasting glycemia [IFG]) exists.

**Methods:**

In our cohort of 1442 non-diabetic subjects of European origin (normal glucose tolerance [NGT] n = 1046, isolated IFG n = 142, isolated IGT n = 140, IFG+IGT n = 114), an impact on glucose homeostasis has been shown for 9 SNPs in previous studies in this specific cohort. We analyzed these SNPs (within or in the vicinity of the genes *TCF7L2*, *KCNJ11*, *HHEX*, *SLC30A8*, *WFS1*, *KCNQ1*, *MTNR1B*, *FTO*, *PPARG*) for association with prediabetes.

**Results:**

The genetic risk load was significantly associated with the risk for IGT (p = 0.0006) in a model including gender, age, BMI and insulin sensitivity. To further evaluate potential confounding effects, we stratified the population on gender, BMI and insulin sensitivity. The association of the risk score with IGT was present in female participants (p = 0.008), but not in male participants. The risk score was significantly associated with IGT (p = 0.008) in subjects with a body mass index higher than 30 kg/m^2^ but not in non-obese individuals. Furthermore, only in insulin resistant subjects a significant association between the genetic load and the risk for IGT (p = 0.01) was found.

**Discussion:**

We found that T2D genetic risk alleles cause an increased risk for IGT. This effect was not present in male, lean and insulin sensitive subjects, suggesting a protective role of beneficial environmental factors on the genetic risk.

## Introduction

The etiology of type 2 diabetes (T2D) is multifactorial, because it arises from a complex interaction between environmental factors and genetic susceptibility [1]. The major environmental causes are sedentary lifestyle and high energy intake leading to obesity and insulin resistance. Genetic susceptibility is determined by a multitude of genes contributing to the overall predisposition, each gene having a rather small individual effect [2], [3]. Most of the approximately 40 known genetic variants conferring increased risk for T2D have been discovered by genome-wide association studies (GWAS) [4]. With this method, associations between genomic variants and diabetes prevalence or quantitative glycemic traits like increased fasting plasma glucose or 2-hour plasma glucose can be established [5]. Most discovered diabetes-risk variants have a predominant effect on insulin secretion, and there are only few that markedly influence body adiposity and insulin sensitivity [2].

The natural history of type 2 diabetes (T2D) includes hyperglycemic states preceding the manifestation of overt diabetes [6], [7]. Only 50% of individuals with IGT progress to diabetes over their lifetime and the annual progression rate vary from 2.3 to 11% [8]. Impaired glucose tolerance (IGT) is independently associated with an elevated risk for atherosclerotic vascular diseases [9]–[11].It has been shown that summation of known diabetes-risk alleles increases the incidence of T2D [12], [13]. However, it is unknown whether previously identified diabetes risk genes can also determine risk for prediabetes. Several genetic variants have been identified in GWAS which associate with fasting glucose or postload glucose after OGTT. To our knowledge, discrete categories of prediabetes have not yet been tested in association with a genetic risk score. We therefore aimed to answer the question whether known genetic variants bearing susceptibility for diabetes can also determine risk for prediabetes (IFG or IGT). We investigated 9 single nucleotide polymorphisms (SNPs) which were previously shown to associate either with insulin sensitivity or with insulin secretion in our study population. The examined SNPs were rs7903146 in *TCF7L2*, rs7923837 in *HHEX*, rs13266634 in *SLC30A8*, rs1001013 in *WFS1*, rs5219 in *KCNJ11*, rs151290 in *KCNQ1*, rs10830963 in *MTNR1B*, rs8050136 in *FTO* and rs1808282 in *PPARG,* as genotyped for previous investigations [14]–[20]. Seven of these SNPs primarily modulate insulin secretion (*TCF7L2*, *HHEX*, *SLC30A8*, *WFS1*, *KCNJ11*, *KCNQ1*, *MTNR1B*).

## Methods

### Subjects

We studied 1442 non-diabetic persons with European ancestry who were selected from the on-going TUEbingen Family study (TUEF). Data of this study population have been used in previous publications [14]–[20], but a genetic risk score was not tested for prediabetes as outcome parameter in this population before. Up to now, more than 2000 individuals who are at increased risk of T2D have participated in the study. Increased risk of diabetes was defined as family history of type 2 diabetes, BMI >27 kg/m^2^ or prior diagnosis of IGT. Most of the participants were recruited by newspaper advertisements. Participants were first interviewed on telephone. Those who fulfilled the inclusion criteria were invited to visit the test facility of the university hospital. Informed written consent was obtained from all participants and the Ethics Committee of the medical faculty of the University of Tübingen approved the protocol. Anthropometric parameters and blood pressure were measured. A 12-lead ECG test was performed. A physician obtained detailed medical history and performed a physical examination. Persons with symptomatic cardiovascular or endocrine disease, abnormal ECG or serious chronic disease, judged at the discretion of the attending physician, were excluded from the study. After excluding participants who turned out to have diabetes, selection was done based on the availability of the investigated demographic parameters and genotype data. A positive family history of diabetes was ascertained if at least one first-degree relative had diabetes. From 1442 individuals, 34 took lipid lowering medications (2%) and 114 took antihypertensive medications (8%).

Obesity was defined as a BMI greater than or equal to 30 kg/m^2^. Information on baseline demographic and glycemic parameters of the cohort is provided in Table 1. The insulin resistant and insulin sensitive subgroups were defined by separating the groups by the median insulin sensitivity index.

**Table 1:** Characteristics of the study population.

	female	male	p	NGT^a^	IFG^b^	p^c^	IGT^d^	p^c^	IFG+IGT^e^	p^c^
	959	483		1046	142		140		114	
age (years)	39.2±12.9	40.2±13.4	0.26	37.5±12.4	43.4±13.0	<0.0001	42.1±13.2	<0.0001	49.7±13.3	<0.0001
BMI (kg/m2)	28.7±8.1	28.1±7.3	0.66	27.2±6.8	32.7±10.8	<0.0001	29.7±7.3	<0.0001	33.8±9.6	<0.0001
family history of diabetes	48.5%	49.0%	0.77	45.8%	58.5%	0.006	60.4%	0.002	52.7%	0.19
systolic blood pressure (mmHg)	121±17	126±16	<0.0001	119±15	128±16	<0.0001	128±16	<0.0001	134.0±18.9	<0.0001
diastolic blood pressure (mmHg)	75±11	77±11	0.07	74±10	78±11	0.002	79±12	<0.0001	81.6±10.5	<0.0001
Fasting glucose (mmol/l)	5.1±0.5	5.2±0.6	0.001	4.9±0.4	5.9±0.3	<0.0001	5.1±0.3	<0.0001	6.1±0.3	<0.0001
2-hour glucose (mmol/l)	6.4±1.6	6.1±1.7	0.001	5.6±1.1	6.2±1.0	<0.0001	8.7±0.8	<0.0001	9.2±1.0	<0.0001
Fasting insulin (pmol/l)	64±54	58±47	0.005	54±43	83±72	<0.0001	71±52	<0.0001	102.4±64.0	<0.0001
2-hour insulin (pmol/l)	453±463	376±412	<0.0001	330±299	424±345	<0.0001	745±580	<0.0001	928±835	<0.0001
HOMA-ß (AU)	139±139	117±86	0.001	131±134	117±104	0.015	151±103	0.004	133±76	0.20
HOMA-IR (AU)	2.5±2.2	2.3±2.1	0.001	2.0±1.6	3.6±3.2	<0.0001	2.7±2.0	<0.0001	4.6±3.1	<0.0001
TG (mmol/l)	1.2±0.8	1.9±2.8	<0.0001	1.3±1.7	1.5±1.7	0.004	1.8±2.1	<0.0001	1.8±1.4	<0.0001
Cholesterin (mmol/l)	5.0±0.9	5.0±1.1	0.45	5.0±1.0	5.0±0.9	0.89	5.2±1.0	0.022	5.1±0.9	0.044
HDL (mmol/l)	1.5±0.4	1.2±0.3	<0.0001	1.4±0.4	1.3±0.3	0.001	1.4±0.3	0.008	1.3±0.3	0.001
LDL (mmol/l)	3.0±0.8	3.2±0.8	0.0003	3.0±0.8	3.1±0.8	0.24	3.2±0.8	0.006	3.2±0.8	0.020
lipid lowering therapy	4.3%	2.4%	0.0007	1.1%	4.9%	0.003	5.7%	0.001	7.0%	0.0002
antihypertensive therapy	9.5%	7.9%	0.12	4.8%	12.7%	0.0007	10.7%	0.009	27.2%	<0.0001

Data are means ± standard deviations.

a normal glucose tolerance.

b isolated impaired fasting glycemia.

c as compared to normal glucose tolerance.

d isolated impaired glucose tolerance.

e concomitant impaired fasting glycemia and impaired glucose tolerance.

### Genotyping

DNA from whole blood was isolated using a commercial DNA isolation kit (NucleoSpin, Macherey& Nagel, Düren, Germany). Genotyping was performed as previously reported [15] [14] [16] using the TaqMan assay (Applied Biosystems, Forster City, CA, USA). The TaqMan genotyping reaction was amplified on a GeneAmp PCR System 7000, and fluorescence was detected on an ABI Prism 7000 sequence detector (Applied Biosystems). The genotypes were verified in 50 randomly selected subjects by bidirectional sequencing, and both methods resulted in 100% identical results. All SNPs obeyed the Hardy-Weinberg equilibrium.

### Classification of Glycemic Conditions and Calculations

Oral glucose tolerance tests (OGTT) were performed as recommended by the World Health Organization [21]. Subjects were classified upon fasting and postload (2-hours) glucose levels during OGTT according to recommendations of the American Diabetes Association. Elevated postload glucose (> = 7.8 mmol/l) with normal fasting glucose (<5.6 mmol/l) is termed isolated IGT (n = 140), and elevated fasting glucose (> = 5.6 mmol/l) with normal postload glucose (<7.8 mmol/l) is termed isolated IFG (n = 142) in this study. Because of the pivotal role of postload hyperglycemia in the development of diabetes [6], the subgroup termed IGT in this study comprises all participants with elevated postload glucose (independently of fasting glycemia) (n = 254). The prediabetes subgroup comprises all participants with IFG and/or IGT (n = 396).

For the estimation of insulin sensitivity, the insulin sensitivity index (ISI) was calculated from glucose and insulin values throughout the OGTT as proposed by Matsuda and DeFronzo [22] with the following equation (g denotes glucose, i denotes insulin levels at specific OGTT time-points):
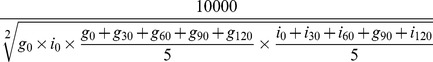



### Risk Scores

Each SNP was coded as the number of diabetes risk alleles from 0 to 2. Summation of the amount of risk alleles yielded a simple risk score. The **simple (unweighted) risk score** ρ’_j_ (for the participant j) was calculated as follows:
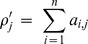
We additionally calculated a weighted risk score with a method described earlier by others [23], [24]. In short, the number of per-SNP risk alleles was multiplied by the SNP-specific effect size. Effect size was derived from estimated odds ratios of incident diabetes as found in the literature (see Electronic Table S1). The **risk score** ρ_j_ (weighted risk score for the participant j) was calculated as follows



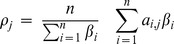
Where


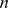
 is the total number of SNPs investigated, in this case 9, and


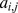
 is the risk allele count for SNP_i_, participant j


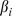
 is the effect size for SNP_i_ which has been calculated as


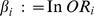
the per allele odds ratio (OR) of SNP_i_ for diabetes as found in the literature (see Table S1).

Note that 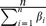 is a constant in this study. Its sole function is to make the weighted risk score easier to interpret, because it is then comparable with the total number of risk alleles (with the simple risk score).

Another risk score that mainly reflects disruption of insulin secretion was constructed from 7 SNPs after omitting the *FTO* and *PPARG* loci.

### Statistical Analysis

Data are given as means ± SD when not stated otherwise. Means were compared with Student’s t test for normally distributed variables and with the Wilcoxon test for non-normally distributed variables. Differences between binary outcomes were tested with Fisher’s exact test. Logistic regression was applied in the analyses of the multivariable models with the genetic risk score. Prediabetes categories were used as dichotomous outcomes in these models. Non-normally distributed variables were transformed to their natural logarithms to approximate normal distribution; p values were obtained from effect likelihood ratio tests. Since there was only one statistical hypothesis to test, and all SNPs included in the risk score were known diabetes SNPs, no correction for multiple testing was necessary. All tests were performed as two-tailed tests, a p<0.05 was considered statistically significant.

Given the distribution of the weighted genetic risk score in the investigated population, our study had sufficient power (1-β = 0.8) to demonstrate a per-allele odds ratio as low as 1.043 for the whole cohort (n = 1440) and an odds ratio of 1.075 for the smallest subgroup studied (n = 456). In order to detect risk score differences with sufficient power (1-β = 0.8), the required effect sizes for the subgroups prediabetes, IGT, isolated IGT and isolated IFG were 0.17, 0.19, 0.25 and 0.25, respectively.

All calculations were done with JMP 8.0 (SAS Institute, Cary, NC, USA), except for sensitivity analyses that were conducted with G*Power Version 3.1.2 [25].

## Results

Seeking to answer the question whether T2D-related genetic risk determines risk for IGT or prediabetes, we summed up weighted risk alleles from 9 established diabetes-risk SNPs for each of the 1442 non-diabetic subjects, and ascertained the clinical at-risk status from an OGTT according to recommendations of the ADA.

The weighted genetic risk score was not associated with fasting glucose after adjustment for gender, age and BMI (p = 0.89). However, it was associated with postload glucose after adjustment for gender, age and BMI (p = 0.002). In logistic regression models including gender, age, BMI and insulin sensitivity, both the simple (p = 0.014) and the weighted sum of risk alleles (p = 0.0006) predicted the presence of IGT. In the model with the weighted genetic risk score, gender did not associate with IGT (β = −0.195 (0 = female, 1 = male), p = 0.26), but age (β = 1.683, p<0.001), BMI (β = −1.337, p = 0.001) and insulin sensitivity (β = −1.85, p<0.01) were significant predictors. In an extended model, variables of lipid metabolism, family history of diabetes and use of lipid lowering and antihypertensive drugs were added as covariates. Since LDL and total cholesterol were highly correlated, adjustment was carried out for LDL, HDL and triglycerides, but total cholesterol was not included. In this comprehensive model, the weighted genetic risk score associated with IGT (β = 0.15, p = 0.0003) after adjustment for gender, age, BMI, insulin sensitivity, LDL, HDL, triglycerides, use of lipid lowering drugs, use of antihypertensives and family history of diabetes.

When testing for prediabetes or for isolated IGT, the weighted risk score showed a significant association with both outcomes (p = 0.045 and 0.017, respectively), which was not significant when using the simple risk score. However, even the simple, unweighted risk score associated well with the risk for prediabetes when it was limited to the alleles of the seven genes involved in impaired insulin secretion (p = 0.0034). Isolated IFG did not show any significant correlation with the genetic risk (p = 0.18 with the weighted risk score).

We furthermore tested associations of the T2D-related genetic risk with the presence of prediabetes in female/male, obese/non-obese and insulin resistant/sensitive strata of the cohort.

The weighted risk score adjusted for age, BMI and insulin sensitivity demonstrated an association with IGT (n = 959, p = 0.008) in females, but failed to associate with IGT in males (n = 483, p = 0.54). Sensitivity analysis yielded a minimal sample size of 293 for showing the effect seen in female participants.

**Figure 1:**
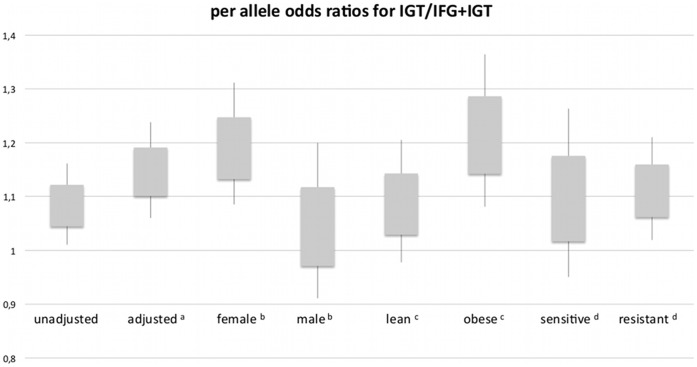
Odds ratios for IGT per risk allele in the whole cohort and in subgroups. Boxes indicate odds ratio ± standard error; whiskers indicate 95% confidence interval. Overall indicates whole cohort. Lean indicates BMI <30 kg/m^2^. Obese indicates BMI > = 30 kg/m^2^. Sensitive indicates insulin sensitivity index > = 14.3. ^a^adjusted for sex, age, BMI and insulin sensitivity; ^b^ adjusted for age, BMI and insulin sensitivity; ^c^ adjusted for sex, age and insulin sensitivity; ^d^ adjusted for sex, age and BMI.

For the obese subjects of the cohort (n = 456), a significant association was found between the sum of weighted risk alleles and the prevalence of IGT (p = 0.008). This association was not present in non-obese subjects (n = 986, p = 0.37). When investigating insulin resistant subjects who had lower than median insulin sensitivity indices (n = 715), a significant association was found between the weighted risk score and the prevalence of IGT (p = 0.017). This association was not present in insulin sensitive subjects (n = 714, p = 0.2). These results are visualized with per-allele odds ratios for IGT in Figure 1.

## Discussion

In the present study, we asked whether the summation of risk alleles known to be associated with diabetes is already associated with prediabetic states. It is worthwhile to pose this question because the genetic risk for impairing glycemia may become relevant only when impaired glucose tolerance progresses to diabetes, but not when normal glucose tolerance progresses to impaired glucose tolerance. This has been shown, e.g. for SNPs in *TCF7L2* and *WFS1* which become more and more relevant during the progression of prediabetes stages towards clinically overt type 2 diabetes [14]. We decided to sum up all relevant risk genes that associated with impaired insulin secretion, impaired insulin sensitivity or both in our specific cohort. This approach is a powerful way to test associations between genetic markers and clinical outcome, because the genetic risk score can integrate synergistic effects of individual markers.

We showed that the risk score calculated from 9 important diabetes-related SNPs is similarly associated with a prediabetic state of impaired glucose metabolism. This effect was independent of well-known environmental risk factors like age, body weight and insulin resistance. The finding suggests that genetically determined risk for diabetes is involved in the very early transition from normal glucose tolerance to impaired glucose tolerance.

Surprisingly BMI was negatively associated with the dichotomous outcome parameter IGT in the model adjusted for insulin sensitivity. The association of BMI with IGT was positive when insulin sensitivity was omitted from the covariates. This suggests that once controlling for insulin sensitivity, higher BMI becomes protective against IGT. A possible explanation for this finding could be a statistical dissociation of BMI into metabolically harmful and metabolically non harmful body mass by adjustment for insulin sensitivity. The term “metabolically benign obesity” describes persons with elevated body mass and normal insulin sensitivity [26]–[28]. It is possible that in the non-diabetic healthy population of the present study with a high proportion of obese subjects (32% have BMI >30 kg/m^2^), subjects with benign obesity were overrepresented which could bias the results of the association analysis.

It is well known that obesity and insulin sensitivity have a modulatory effect on the detection of genetic susceptibility for T2D [29], [30]. For example, we showed that the effect of diabetes risk SNPs on insulin secretion is only apparent in insulin resistant individuals. Furthermore, the work of Cornelis et al suggested that a genetic risk score had a stronger effect in predicting diabetes among obese persons [31]. When stratifying our population in obese and non-obese participants as well as in insulin resistant and insulin sensitive participants, we found that the risk alleles associate with prediabetes only in the obese and insulin resistant groups. The effect of the genetic risk score was not present in non-obese and insulin sensitive subjects, which suggests that leanness and insulin sensitivity may protect from the genetic risk. Taken together, these findings propose that not only the manifestation of diabetes but also the manifestation of prediabetes is a result of an interaction between environment and inherited susceptibility. This will have to be confirmed in longitudinal studies.

Although sexual dimorphism in the effect of genetic loci on waist-hip-ratio has already been identified [32], up to now there is only limited data on diabetes-genes. In our study population there were considerably less males than females. Sensitivity analysis suggests that the lack of effect in male participants cannot be solely explained by smaller subgroup size. In populations of European origin, IGT is generally more prevalent among women compared to men [33]. Although such a difference was not statistically significant in our cohort, an ascertainment bias may have played a role. Since about three-quarters of female participants in this study were younger than 50 years, interaction of the investigated genetic risk with sex hormones is a hypothetical possibility which could be investigated in future studies. Observational data support a connection between diabetes and sex-hormones [34]. For example, sexual hormone binding globulin (SHBG) levels were negatively associated with diabetes [34], [35] and diabetes related traits [36], and studies utilizing mendelian randomization with the *SHBG* gene provide evidence for a causal link [37].

Restricting the investigated risk load on the genetic variants related mainly to insulin secretion (7 out of 9 SNPs) did not abolish, but rather strengthen the association with prediabetes. One may therefore speculate that the genetic risk for prediabetes is mediated mainly through the genetic variation affecting insulin secretion.

From the categories of increased risk for diabetes, IGT was most consistently associated with genetic risk. Testing isolated IFG or IGT groups deleted some of the associations. This may be caused by the fact that IGT has stronger associations with the risk genes tested here than IFG, or simply by low statistical power when testing the smaller isolated IGT and IFG groups separately.

This study is limited by several factors. Out of approximately 40 known diabetes-related variants only 9 were tested. The fact that our cohort is not population-based, the relatively large prevalence of prediabetic conditions among participants (28%) and the female predominance could probably have led to some recruiting bias. Yet our study provides a proof of concept that IGT can be determined by a genetic risk score. It was demonstrated that the weighted sum of alleles that associate with an increased risk for manifest diabetes also predicts an increased risk for IGT. This effect was mainly restricted to obese and insulin resistant individuals and is not present in lean and insulin sensitive subjects, suggesting a modulating role of these factors on the genetic risk. In the future, intensive lifestyle intervention to pre-empt and treat obesity could be preferentially targeted on those with higher genetic risk to yield greater efficiencies in preventing diabetes.

## Supporting Information

Table S1 **Calculation of the genetic risk score.** ^a^For the FTO gene, rs8050136 was genotyped which is in complete linkage disequilibrium with rs9939609.
(DOC)

## References

[1] Ershow A (2009). Environmental influences on development of type 2 diabetes and obesity: challenges in personalizing prevention and management.. Journal of diabetes science and technology.

[2] Staiger H, Machicao F, Fritsche A, Haring H-U (2009). Pathomechanisms of Type 2 Diabetes Genes.. Endocr Rev.

[3] Doria A, Patti M-E, Kahn CR (2008). The emerging genetic architecture of type 2 diabetes.. Cell Metab.

[4] McCarthy MI (2010). Genomics, Type 2 Diabetes, and Obesity.. N Engl J Med.

[5] Billings L, Florez J (2010). The genetics of type 2 diabetes: what have we learned from GWAS?. Annals of the New York Academy of Sciences.

[6] Tabák A, Jokela M, Akbaraly T, Brunner E, Kivimäki M (2009). Trajectories of glycaemia, insulin sensitivity, and insulin secretion before diagnosis of type 2 diabetes: an analysis from the Whitehall II study.. Lancet.

[7] Weir GC, Bonner-Weir S (2004). Five stages of evolving beta-cell dysfunction during progression to diabetes.. Diabetes.

[8] DeFronzo RA, Abdul-Ghani M (2011). Type 2 diabetes can be prevented with early pharmacological intervention.. Diabetes Care.

[9] DECODE Study Group (2003). Is the current definition for diabetes relevant to mortality risk from all causes and cardiovascular and noncardiovascular diseases?. Diabetes Care.

[10] Rydén L, Standl E, Bartnik M, Van den Berghe G, Betteridge J (2007). Guidelines on diabetes, pre-diabetes, and cardiovascular diseases: executive summary. The Task Force on Diabetes and Cardiovascular Diseases of the European Society of Cardiology (ESC) and of the European Association for the Study of Diabetes (EASD). Eur.. Heart J.

[11] Ford ES, Zhao G, Li C (2010). Pre-diabetes and the risk for cardiovascular disease: a systematic review of the evidence. J. Am. Coll.. Cardiol.

[12] Lango H, Palmer CNA, Morris AD, Zeggini E, Hattersley AT (2008). Assessing the combined impact of 18 common genetic variants of modest effect sizes on type 2 diabetes risk.. Diabetes.

[13] Reiling E, van ’t Riet E, Groenewoud MJ, Welschen LMC, van Hove EC (2009). Combined effects of single-nucleotide polymorphisms in GCK, GCKR, G6PC2 and MTNR1B on fasting plasma glucose and type 2 diabetes risk.. Diabetologia.

[14] Heni M, Ketterer C, Thamer C, Herzberg-Schäfer SA, Guthoff M (2010). Glycemia determines the effect of type 2 diabetes risk genes on insulin secretion.. Diabetes.

[15] Kirchhoff K, Machicao F, Haupt A, Schäfer S, Tschritter O (2008). Polymorphisms in the TCF7L2, CDKAL1 and SLC30A8 genes are associated with impaired proinsulin conversion.. Diabetologia.

[16] Staiger H, Stancáková A, Zilinskaite J, Vänttinen M, Hansen T (2008). A candidate type 2 diabetes polymorphism near the HHEX locus affects acute glucose-stimulated insulin release in European populations: results from the EUGENE2 study.. Diabetes.

[17] Schäfer SA, Müssig K, Staiger H, Machicao F, Stefan N (2009). A common genetic variant in WFS1 determines impaired glucagon-like peptide-1-induced insulin secretion.. Diabetologia.

[18] Müssig K, Staiger H, Machicao F, Kirchhoff K, Guthoff M (2009). Association of type 2 diabetes candidate polymorphisms in KCNQ1 with incretin and insulin secretion.. Diabetes.

[19] Staiger H, Machicao F, Schäfer SA, Kirchhoff K, Kantartzis K (2008). Polymorphisms within the novel type 2 diabetes risk locus MTNR1B determine beta-cell function.. PLoS ONE.

[20] Haupt A, Thamer C, Staiger H, Tschritter O, Kirchhoff K (2009). Variation in the FTO gene influences food intake but not energy expenditure. Exp. Clin. Endocrinol.. Diabetes.

[21] Alberti KG, Zimmet PZ (1998). Definition, diagnosis and classification of diabetes mellitus and its complications. Part 1: diagnosis and classification of diabetes mellitus provisional report of a WHO consultation. Diabet..

[22] Matsuda M, DeFronzo RA (1999). Insulin sensitivity indices obtained from oral glucose tolerance testing: comparison with the euglycemic insulin clamp.. Diabetes Care.

[23] Hivert M-F, Jablonski KA, Perreault L, Saxena R, McAteer JB (2011). Updated genetic score based on 34 confirmed type 2 diabetes Loci is associated with diabetes incidence and regression to normoglycemia in the diabetes prevention program.. Diabetes.

[24] Meigs JB, Shrader P, Sullivan LM, McAteer JB, Fox CS (2008). Genotype score in addition to common risk factors for prediction of type 2 diabetes. N. Engl.. J. Med.

[25] Faul F, Erdfelder E, Buchner A, Lang A-G (2009). Statistical power analyses using G*Power 3.1: tests for correlation and regression analyses.. Behav Res Methods.

[26] Brochu M, Tchernof A, Dionne IJ, Sites CK, Eltabbakh GH (2001). What are the physical characteristics associated with a normal metabolic profile despite a high level of obesity in postmenopausal women? J. Clin. Endocrinol.. Metab.

[27] Sims EA (2001). Are there persons who are obese, but metabolically healthy? Metab. Clin.. Exp.

[28] Stefan N, Kantartzis K, Machann J, Schick F, Thamer C (2008). Identification and characterization of metabolically benign obesity in humans. Arch. Intern. Med.. http://dx.doi.org/10.1001/archinte.168.15.1609.

[29] Cauchi S, Nead KT, Choquet H, Horber F, Potoczna N (2008). The genetic susceptibility to type 2 diabetes may be modulated by obesity status: implications for association studies. BMC Med.. Genet.

[30] Haupt A, Guthoff M, Schäfer SA, Kirchhoff K, Machicao F (2009). The inhibitory effect of recent type 2 diabetes risk loci on insulin secretion is modulated by insulin sensitivity. J. Clin. Endocrinol.. Metab.

[31] Cornelis MC, Qi L, Zhang C, Kraft P, Manson J (2009). Joint effects of common genetic variants on the risk for type 2 diabetes in U.S. men and women of European ancestry. Ann. Intern.. Med.

[32] Heid IM, Jackson AU, Randall JC, Winkler TW, Qi L (2010). Meta-analysis identifies 13 new loci associated with waist-hip ratio and reveals sexual dimorphism in the genetic basis of fat distribution. Nat. Genet.. http://dx.doi.org/10.1038/ng.685.

[33] Mykkänen L, Laakso M, Uusitupa M, Pyörälä K (1990). Prevalence of Diabetes and Impaired Glucose Tolerance in Elderly Subjects and Their Association With Obesity and Family History of Diabete.. Diabetes care.

[34] Ding EL, Song Y, Malik VS, Liu S (2006). Sex differences of endogenous sex hormones and risk of type 2 diabetes: a systematic review and meta-analysis.. JAMA.

[35] Ding EL, Song Y, Manson JE, Hunter DJ, Lee CC (2009). Sex hormone-binding globulin and risk of type 2 diabetes in women and men. N. Engl. J. Med.. http://dx.doi.org/10.1056/NEJMoa0804381.

[36] Peter A, Kantartzis K, Machann J, Schick F, Staiger H (2010). Relationships of Circulating Sex Hormone–Binding Globulin With Metabolic Traits in Humans.. Diabetes.

[37] Perry JRB, Weedon MN, Langenberg C, Jackson AU, Lyssenko V (2010). Genetic evidence that raised sex hormone binding globulin (SHBG) levels reduce the risk of type 2 diabetes. Hum. Mol. Genet.. http://dx.doi.org/10.1093/hmg/ddp522.

