# Supplementary material for: Allele Summation of Diabetes Risk Genes Predicts Impaired Glucose Tolerance in Female and Obese Individuals
Source: PLoS One. 2012 Jun 29;7(6):e38224. doi: 10.1371/journal.pone.0038224 (PMC3387191; doi:10.1371/journal.pone.0038224)
Supplement: Table S1 — Calculation of the genetic risk score. aFor the FTO gene, rs8050136 was genotyped which is in complete linkage disequilibrium with rs9939609. (DOC) [file pone.0038224.s001.doc]

## Supplementary Table S1

Calculation of the genetic risk score

The following odds ratios (OR) available in the literature were used for weighting the risk alleles.

| **Gene** | **SNP** | **OR** | **Reference** |
| --- | --- | --- | --- |
| TCF7L2 | rs7903146 | 1.37 | Zeggini 2008 |
| PPARg | rs1801282 | 1.18 | Zeggini 2008 |
| WFS1 | rs10010131 | 1.11 | Zeggini 2008 |
| SLC30A8 | rs13266634 | 1.15 | Zeggini 2008 |
| HHEX | rs1111875 | 1.17 | Zeggini 2008 |
| MTNR1B | rs10830963 | 1.09 | Prokopenko 2009 |
| FTO | rs9939609^a^ | 1.15 | Zeggini 2008 |
| KCNJ11 | rs5219 | 1.16 | Saxena 2007 |
| KCNQ1 | rs151290 | 1.34 | Yasuda 2008 |

^a^For the FTO gene, rs8050136 was genotyped which is in complete linkage disequilibrium with rs9939609.

**References:**

Zeggini E, Scott LJ, Saxena R, et al. (2008) Meta-analysis of genome-wide association data and large-scale replication identifies additional susceptibility loci for type 2 diabetes. Nat Genet 40: 638–645.

Prokopenko I, Langenberg C, Florez JC, et al. (2009) Variants in the melatonin receptor 1B gene (MTNR1B) influence fasting glucose levels. Nat Genet 41: 77–81.

Saxena R, Voight BF, Lyssenko V, et al. (2007) Genome-Wide Association Analysis Identifies Loci for Type 2 Diabetes and Triglyceride Levels. Science.

Yasuda K, Miyake K, Horikawa Y, et al. (2008) Variants in KCNQ1 are associated with susceptibility to type 2 diabetes mellitus. Nat Genet 40: 1092–1097.
